# Direct costs of illness of patients with chronic cough in rural Malawi—Experiences from Dowa and Ntchisi districts

**DOI:** 10.1371/journal.pone.0225712

**Published:** 2019-12-31

**Authors:** Junious M. Sichali, Jahangir A. K. Khan, Elvis M. Gama, Hastings T. Banda, Ireen Namakhoma, Grace Bongololo, Rachael Thomson, Berthe Stenberg, S. Bertel Squire

**Affiliations:** 1 Research for Equity and Community Health (REACH) Trust, Lilongwe, Malawi; 2 Centre for Applied Health Research and Delivery (CAHRD), Liverpool School of Tropical Medicine, Liverpool, United Kingdom; 3 LHL’s International Tuberculosis Foundation (LHL International), Oslo, Norway; The University of Georgia, UNITED STATES

## Abstract

**Introduction:**

Chronic cough is a distressing symptom and a common reason for people to seek health care services. It is a symptom that can indicate underlying tuberculosis (TB) and/or chronic airways diseases (CAD) including asthma, chronic obstructive pulmonary disease (COPD) and bronchiectasis. In developing countries including Malawi, provision of diagnostic services and clinical management of CAD is rudimentary, so it is thought that patients make costly and unyielding repeated care-seeking visits. There is, however, a lack of information on cost of illness, both direct and indirect, to patients with chronic cough symptom. Such data are needed to inform policy-makers in making decisions on allocating resources for designing and developing the relevant health care services to address universal coverage programmes for CAD. This paper therefore explores health seeking costs associated with chronic cough and explores information on usage of the coping mechanisms which indicate financial hardship, such as borrowing and selling household assets.

**Methods:**

This economic study was nested within a community-based, population-proportional cross-sectional survey of 15,795 individuals aged 15 years and above, in Dowa and Ntchisi districts. The study sought to identify individuals with symptoms of chronic airways disease whose health records documented at least one of the following diagnoses within the previous year: TB, Asthma, COPD, Bronchitis and Lower Respiratory Tract Infection (LRTI). We interviewed these chronic coughers to collect information on socioeconomic and socio-demographic characteristics, health care utilization, and associated costs of care in 2015. We also collected information on how they funded their health seeking costs.

**Results:**

We identified 608 chronic coughers who reported costs in relation to their latest confirmed diagnosis in their hand-held health record. The mean care-seeking cost per patient was US$ 3.9 (95% CI: 3.00–5.03); 2.3 times the average per capita expenditure on health of US$ 1.69. The largest costs were due to transport (US$ 1.4), followed by drugs (US$ 1.3). The costs of non-medical inputs (US$ 2.09) was considerable (52.3%). Nearly a quarter (24.4%) of all the patients reportedly borrowed or/and sold assets/property to finance their healthcare. CCs with COPD and LRTI had 85.6% and 62.0% lower chance of incurring any costs compared with the TB patients and any patients with comorbidity had 2.9 times higher chance to incur any costs than the patients with single disease. COPD, Bronchitis and LRTI patients had 123.9%, 211.4% and 87.9% lower costs than the patients with TB. The patients with comorbidity incurred 53.9% higher costs than those with single disease.

**Conclusions:**

The costs of healthcare per chronic cougher was mainly influenced by the transport and drugs costs. Types of diseases and comorbidity led to significantly different chances of incurring costs as well as difference in magnitude of costs. The costs appeared to be unaffordable for many patients.

## Introduction

Tuberculosis and chronic airway diseases (CAD) including asthma, chronic obstructive pulmonary disease (COPD) and bronchiectasis represent a major disease burden to most developing countries, particularly in Sub-Saharan Africa [1–4]. COPD and asthma are estimated to affect 64 million and 235 million people worldwide, respectively [3]. A diagnosis for people with chronic or persistent cough is usually delayed because of individual and health system barriers. Unfortunately, delayed diagnosis and treatment of TB, COPD and asthma facilitates further TB transmission, severity of disease with complications and increased mortality. In the latest estimates by World Health Organisation, COPD was the fourth leading cause of deaths in 2004, and in 2030, it is projected to become the third leading cause, with 5.8 million or 8.6% of the total deaths [3].

It has been estimated that, asthma morbidity and mortality accounts for around 1% of all disability-adjusted life years (DALYs), equivalent to 16 million DALYs lost per year worldwide [3]. A crucial point is that all respiratory diseases, if not diagnosed, treated and managed promptly and correctly, are problematic for individuals and health systems alike [5].

Furthermore, COPD is characterized by airflow limitation that is not fully reversible but nonetheless treatable. The World Health Organization (WHO) estimates that 65 million people have moderate to severe COPD with 5% of all (3 million) global deaths being attributed to COPD [6]. About 90% of these COPD deaths occur in low-income and middle-income countries. At the other end of the spectrum, asthma, a chronic inflammatory disorder of the airways, is characterized by episodes of reversible breathing problems (that may be life-threatening) due to airway narrowing and obstruction. These episodes are characterized by coughing, wheezing, chest tightness and shortness of breath. The frequency and severity of these episodes can have a marked impact on the livelihoods and the well-being of those affected [7]. Bronchiectasis is a severe, chronic infection of the lung associated with permanent abnormal dilatation of bronchi [8]. As a result, mucus clearance is impaired, leading to accumulation of secretions, bacterial overgrowth and recurrent infections.

Health seeking costs associated with chronic cough (CC) are estimated to rank as one of the highest among chronic diseases due to the significant health care utilisation associated with these conditions [9,10]. Severe and poorly controlled chronic cough is believed to have a high economic burden both directly (hospitalisation, diagnostic tests and medicines) and indirectly (productivity loss) [9–12]. In addition, chronic cough is also associated with the loss of future potential earnings related to morbidity and mortality. `

Numerous studies have been published evaluating health seeking costs of CC in high income countries [13–16, 17] however, a dearth of evidence exists on similar studies in low income countries [9, 18–20]. In order to address the major CC challenges that threaten health and economies alike in low income settings, it is important to have information on the type and extent of costs incurred by different groups of individuals affected by chronic cough [9, 21–25].

This is important given that in low income settings, relatively small costs on health can be disastrous and lead to catastrophic healthcare expenditure [10–12]. This implies that the cost of regular chronic cough diagnosis and treatment could be unaffordable to families in low income settings. One challenge that policy makers face is the lack of cost data to inform the design and provision of universal coverage programs for core health service responses to chronic cough [9, 10 and 12]

## Objectives

The main objective of this paper is to explore health seeking costs incurred by chronic coughers starting from the onset of symptoms, to diagnosis and the management of the illness. This is the cost of the whole care-seeking process which includes pre-diagnostics and diagnostic costs, treatment costs, hospitalisation costs as well as guardians (accompanied persons) costs. To reach the main objective, we set out to estimate the direct costs (medical and non-medical) associated with chronic cough, estimate variations in these costs across socio-economic and socio-demographic groups and to identify coping mechanisms with detrimental effects on economic status of the chronic coughers.

## Methods

### Study design

This study was reviewed and approved by The College of Medicine Research and Ethics Committee (COMREC), which is situated within The University of Malawi, College of Medicine. This is a local Institutional Review Board which approves research projects in Malawi. This was a trial with the following approval number: P.07/13/1424 [17]. Participants signed consent forms after agreeing to participate in the study on the basis of information provided by the data collectors. For those less than 18 years, we obtained consent from parents first, before seeking their individual consent and they signed consent form afterwards.

This cross-sectional community-based survey was conducted in 2015 (January to June) in rural areas of two districts, namely, Dowa and Ntchisi, in the central region of Malawi. A total of 15,795 individuals were included in the survey where household was the unit of approach. Among other goals, this survey intended to identify individuals at age 15 years and above with evidence or symptoms of chronic cough/wheeze, and a diagnosis in their health passport. It was also intended to collect data on health seeking costs that chronic coughers incur before getting a diagnosis, after diagnosis, treatment, hospitalisation as well as guardian costs and how health seeking costs incurred by chronic coughers were financed. i.e. financial coping mechanisms.

### Data collection

We administered a validated patient cost questionnaire recommended by the Stop TB Partnership [26] using pre-programmed smartphones. These participants were selected because they reported costs in relation to their latest confirmed diagnosis through health card/passport. Participants with chronic cough were asked to detail their costs including out-of-pocket medical expenditure (for example administration fees, charges for tests, cost of travel to health facilities and food during a long trip and at the health facility) and coping mechanisms (money they borrowed or received from selling assets to cover the cost of diagnosis).

### Data analysis

Data analysis was done in STATA v13.1. Direct medical and non-medical costs associated with chronic cough were summarised using means accompanied by their 95% confidence intervals. An estimate of variations in costs based on socio-demographic and socio-economic status was done by looking at how much each category (age, sex, education level and occupation) of chronic coughers paid to access healthcare. For coping mechanisms, we calculated the absolute number of chronic coughers who sold assets and/or borrowed money to finance health seeking costs. Costs were reported in United States Dollar (US$) using April 2015 exchange rate (US$1 = MK450) from OANDA—https://www.oanda.com/currency/converter/. We collected information on how much was spent for the preceding 12 months at the time of the survey for the utilized health services e.g., pre-diagnostics and diagnostic costs, treatment costs, hospitalisation costs as well as guardian costs. The specific items costed include administrative, laboratory, X-ray, drugs, travel, food as well as accommodation.

Two-part regression analysis was applied to estimate the effect of individuals’ sociodemographic characteristics on the direct healthcare costs for seeking healthcare due to chronic cough. The direct costs was a limited dependent variable and was continuous over most of its distribution but had a mass of observations at zero values. The decision of spending for healthcare and its magnitude might not be statistically independent [27, 28] Applying an Ordinary Least Square (OLS) estimation method of regression coefficient to only those individuals who spent for healthcare might result in selection bias [28]. We thus applied a two-part regression model [27, 29]. The first part estimated the likelihood of incurring any healthcare costs, where 0 meant ‘no cost’ and 1 meant ‘any incurred cost’. This was incorporated in the two-part model with a logit function. The second part considered the magnitude of the direct healthcare costs. An ordinary least square function was applied to predict it. Thus the two-part model used information on both the probability and magnitude of direct costs for healthcare simultaneously in assessing the effects of sociodemographic factors of the individuals [30, 31].

## Results

### Health care costs

There were a total of 608 people, with confirmed diagnosis of CAD in their health card/passport who reported costs in relation to their health care seeking, with a total mean cost of US $3.9 as illustrated in Table 1.

**Table 1:** Mean health care costs (95% CI) of chronic coughers who reported costs and had confirmed diagnosis in health passport (N = 608).

Type of direct costs	Cost component	Mean (US$)	95% CI	Share %	Median (US$)	Minimum (US$)	Maximum (US$)
**Medical**	Administration	0.3	0.14–0.53	46.7	0.2	0.03	51.1
	Diagnostic tests	0.2	-0.00–0.39		2.2	0.02	55.6
	X-ray	0.05	0.01–0.10		4.4	0.1	7.8
	Drugs	1.3	0.83–1.81		3.3	0.09	110
Sub-total		**1.9**	**1.60–2.58**		0.3	0.02	144.4
**Non-medical**	Transport	1.4	1.10–1.74	52.3	2.7	0.02	111.1
	Food	0.6	0.40–0.74		0.7	0.02	42.2
	Accommodation	0.09	-0.00–0.20		6.7	2.2	26.7
Sub-total		**2.09**	**1.61–2.58**		2.2	0.02	113.3
**Total cost**		**3.9**	**3.00–5.03**	**100.0**	**1.8**	**0.02**	**188.9**

Table 1 above provides an overview of the mean costs and 95% confidence intervals by type of direct costs, both medical and non-medical. These costs have been disaggregated into administration, diagnostic tests, X-ray, drugs, transport, food and accommodation for chronic coughers with a confirmed diagnosis in their health passport. Of the total costs incurred by chronic coughers, drugs and transport form the largest part of the expenditure as evidenced by US$ 1.3 and US$ 1.4 respectively. We have compared the health care costs of chronic coughers with the household consumption expenditure in rural Malawi. According to the Malawi Integrated Household Survey of 2010/11, the annual per capita consumption of US$ 107.4 or per household US$ 494 considering the average household size of 4.6 and inflation rate of 23.4% per year during 2012–15 [32, 33]. Our estimated health care costs (US$ 3.9) of chronic coughers thus constituted 0.42% of household consumption expenditure. The mean cost was compared against the household consumption expenditure because we wanted to see how much of the total household consumption expenditure goes to health care seeking.

The magnitude of costs was also dependent on the kind of diagnoses such that some diseases were costlier than others. For instance, Table 2 shows that TB patients incurred the largest average costs ($10.5), followed by the patients with comorbidity ($ 9.4) and Asthma ($ 6.9) respectively. High costs were a result of transport which patients incur in the course of seeking diagnosis and getting treatment but also drugs.

**Table 2:** Costs of chronic coughers based on diagnosis.

Diagnosis	Observations	Mean(US$)	95% CI
TB	17	10.5	-0.72–21.77
Asthma	125	6.9	3.54–10.42
COPD	35	1.1	0.05–2.16
Bronchiectasis	52	0.8	0.21–1.39
LRTI	347	2.9	1.85–3.87
Comorbidity	32	9.4	2.30–16.58
**Total**	**608**	**3.9**	**3.00–5.03**

Further analysis shows that there were also variations in costs incurred by chronic coughers with respect to symptoms such that those with breathlessness paid slightly higher costs than those with other symptoms as seen from Table 3.

**Table 3:** Costs of care by symptoms of the chronic coughers.

Symptoms	Observations	Mean (US$)	95% CI
Cough	289	3.6	2.52–4.69
Cough and blood	48	7.1	2.3–11.91
Wheeze	190	7.0	4.23–9.81
Breathlessness	197	7.1	4.48.– 9.79
Sputum	168	4.4	2.37–6.48

multiple signs are possible

It was also observed that there were variations in costs with respect to sociodemographic and socioeconomic conditions of the chronic coughers (Table 4). The results showed that men had higher costs (US$ 4.2) than women (US$ 3.9), but the difference was not statistically significant. This difference can also be explained by the fact that most women have less money to spend as such they cannot afford the higher health care seeking costs. However, health seeking costs increased with age such that those aged 65 and above incurred higher costs (US$ 6.5) than those aged below 65 years. Marital status was also a cost determinant such that widows incurred higher cost (US$ 5.5) than those who are married (US$ 4.2) and divorced (US$ 3.1).

**Table 4:** Costs of care considering socio-demographics of chronic coughers.

Sex	Observations	Mean (US$)	(95% CI)
Male	221	4.2	2.08–6.32
Female	387	3.9	2.78–4.97
**Age (years)**			
15–19	48	2.0	0.94–3.13
20–49	363	3.6	2.52–4.71
50–64	115	4.2	1.90–6.46
65 and above	82	6.5	1.46–11.63
**Marital status**			
Married	463	4.2	2.87–5.45
Divorced/separated	42	3.1	1.29–4.90
Widowed	43	5.5	1.19–9.81
Never married	60	2.3	1.30–3.23
**Total**	**608**	**3.9**	**3.00–5.03**

Most chronic coughers who reported health seeking costs had primary level education and their cost was slightly higher than those without any formal education and those with secondary education and above (Table 5). Most of them were farmers while some were casual workers, civil servants and the private sector. Civil servants and private sector employees had higher costs, though not statistically significant, than any other occupations probably because they can afford to access health care from the private hospitals which is usually expensive.

**Table 5:** Health care costs of chronic coughers with different socio-economic condition.

Socioeconomic group	Observations	Mean(US$)	(95% CI)
**Educational level**			
No formal education	110	3.7	2.91–5.30
Primary level	413	4.1	2.71–5.54
Secondary and above	85	3.8	2.05–5.64
**Occupation**			
Farming	529	3.9	2.82–5.02
Business	21	1.3	-0.25–2.80
Casual work	17	1.8	0.44–3.11
Civil servant	14	14.7	-3.29–32.78
Private sector	1	10	
Craftsman	1	0	
Remittances	12	3.2	1.19–5.30
Social cash transfer	0	0	

Our two-part multiple regression model found that the presence of diseases and comorbidity led to significantly higher chance of incurring any healthcare costs (stage 1 in Table 6). CCs with COPD and LRTI had 85.6% and 62.0% lower change of incurring any costs compared with the TB patients. Further, any patients with comorbidity had 2.9 times higher chance to incur any costs than the patients with single disease. Stage 2 of the regression model (Table 6) showed that among those who had any healthcare costs, COPD and LRTI patients had 123.9%, 211.4% and 87.9% lower costs than the patients with TB. The patients with comorbidity incurred 53.9% higher costs than those with single disease.

**Table 6:** Determining factors of healthcare costs.

Variables	Description	Dependent variable = Direct costs of healthcare for chronic coughing	
		Stage1. Acquiring cost (logistic model)	Stage2. Log model of direct costs
*Sex* (ref = male)	Female	0.945 (1.216)	0.273 (0.187)
*Age-group (Ref = 15–19 years)*	20–49 years	1.064 (1.625)	0.453 (0.483)
	50–64 years	1.037 (1.705)	0.728 (0.728)
	65 years and above	1.006 (1.774)	0.728 (0.558)
*Marital status (Ref = married)*	Divorced/separated	0.871 (1.415)	-0.132 (0.346)
	Widow	1.335 (1.541)	-0.301 (0.370)
	Never married	0.528 (0.1576)	0.723 (0.454)
*Education Ref = No formal education)*	Primary	1.261 (1.275)	-0.069 (0.230)
	Secondary and above	1.067 (1.434)	0.253 (0.346)
*Occupation (Ref = farmer)*	Business, craftsman	0.528 (1.586)	-0.716 (0.551)
	Civil servant, Private sector	0.777 (1.797)	0.889* (0.554)
	Casual worker, Craftsman, Remittances, Social cash transfer	1.219 (1.577)	-0.357 (0.370)
*Disease (Ref = Tuberculosis)*	Asthma	1.255 (1.777)	-0.551 (0.409)
	COPD	0.144*** (1.914)	-1.239** (0.605)
	Bronchitis	0.505 (1.820)	-2.114*** (0.460)
	LRTI	0.380* (1.726)	-0.879** (0.404)
*Comorbidity (Ref = No)*	Yes	2.889* (1.794)	0.539* (0.329)
Constant		2.464 (2.129)	6.944*** (0.644)
N		595	346
LRchi2(17)		61.97	
Prob. >chi2		0.000	
Pseudo R2		0.077	
F(17, 328)			3.16
Prob. > F			0.000
Adjusted R2			0.096

***, ** and * denote significant at 1%, 5% and 10% risk level respectively.

We, further found that the individuals with occupational attachment to civil service and private sectors had significantly higher costs (88.9%) than the farmers. No other socioeconomic or demographic factors showed any significant influence on costs for healthcare of the CCs.

### Financial coping mechanisms

In addition to income and savings, two major coping mechanisms for health care payments were observed, namely borrowing and selling assets/properties. Our findings in Table 7 below show that some chronic coughers borrowed money from various sources while others sold something including household assets. Most borrowing came from the neighbours (41%) followed by borrowing from family members (33%) and very few (6%) through bank loans.

**Table 7:** Distribution of chronic coughers considering the source of borrowing and items (assets/property) of selling.

Mechanism	Source	Number (N = 87*)	Share (%)
Borrowing	Family	29	33
	Neighbor	36	41
	Bank	5	6
	Cooperative	14	16
	Others	3	3
	**Total**	**87**	**100**
	**Item**	Number (N = 148**)	**Share (%)**
Selling assets/property	Land	1	0.7
	Livestock	45	30.4
	Vehicle	0	0
	Household item	8	5.4
	Farm produce	87	58.8
	Others	7	4.7
	**Total**	**148**	**100**

* One person borrowed from both family and neighbours which means that 86 (not 87) people borrowed because one person was counted on two sources

** 7 people had multiple responses, meaning that 141 people sold any items.

Most chronic coughers sold household assets to cover health care costs and the majority of costs came from selling farm products (58.8%) and livestock (30.4%). See Table 6 above.

## Discussion

A total of 608 chronic coughers reported health care costs in relation to their latest illnesses and the mean total cost was estimated to be US$ 3.9 in a 12-month period. The largest share of these costs was due to transport (US$ 1.4), followed by medicines (US$1.3). Considering that the majority of the population where the study was done is poor, the 0.42% of the total household consumption expenditure is a huge burden. The variations in costs could be described by demographic and socioeconomic characteristics of the investigated chronic coughers. We observed that chronic coughers at age of 65 years and above had the largest costs which reduced with younger ages. This was probably due to the fact that most elderly people required a guardian to go to the health facility where they sought health care and the costs drove upwards mainly due to transport, food and accommodation. We also noted that the costs were higher for widows (US $5.5) than those who were married, divorced/separated and those who had never been married. However, no clear reasons of such disparities could be identified. Our multivariate analysis showed that patients with different diseases had significantly different chance of incurring healthcare costs and that costs varied depending on the type of diseases and well as the presence of comorbidity.

Our findings, however, were consistent with previous studies, which investigated chronic coughers’ pathways to seeking health care and the associated costs they incurred in other low-income settings [1,21,23,25]. As in Uganda, most chronic cough patients followed a complex pathway, including herbalist, informal providers and formal providers [9]. According to our findings and those of previous studies, this complex pathway often resulted in repeated visits to the providers, which possibly contributed to increasing health care costs.

We do not have scope of estimating the incidence of catastrophic costs of households due to limitation of data particularly on household income or consumption expenditure. However, using a proxy measurement of financial protection, this study confirmed that many chronic coughers sold assets/property and borrowed money to pay for health care. Such findings gave an indication that the costs of care, even if not very high in absolute terms, were not affordable for many poor households and might have brought economic impoverishment to some on a temporary basis if it was not persistent.

We were aware of a limitation of this study regarding recall bias. We used a cross-sectional survey covering a 12-month period, which might be subject to recall bias while our main variables of interest dealt with costs of healthcare. Although the bias might be low with the reported costs of the current visits, we suspect that the costs of previous visits might suffer more from this recall bias.

Despite this limitation, the study does present important insights into health seeking costs of chronic coughers and illuminates some coping mechanisms that can cause or perpetuate poverty. First, while selling of assets can help raise money to cover chronic cough related expenses, it is important to note that asset sale involving disposal of factors of production like land may increase the vulnerability of households. Second, borrowing money from family, friends or money- lenders may represent a reasonable trade-off overtime. However, they can increase the economic vulnerability of households in the long term, particularly for large health seeking costs [34].

We propose that further studies on costs of chronic coughers should be done so that costing data can inform policy makers in the design and provision of universal coverage programs for core chronic cough health services. Future studies should also look at management of CAD and TB in relation to cost implication. Addressing major chronic cough challenges that threaten health and economies alike in low-income settings requires information on the type and extent of costs incurred by different groups (income groups, geographic regions etc.) of individuals affected by chronic cough, which can contribute with more knowledge than that found by this current study. The results from the current study, however, can add to the knowledge of the policy makers in designing and provision of universal coverage programs for core chronic cough health services [9, 10 and 12]

## Conclusion

The costs of healthcare per chronic cougher was mainly influenced by the costs of transport and drugs and these costs varied with demographic and socioeconomic characteristics of the patients. Types of diseases and comorbidity led to significantly different chances of incurring costs and the magnitude of costs varied significantly. Though the costs were not reportedly very high as a share of total household consumption expenditure, it appeared to be unaffordable for many patients.

## Supporting information

S1 Data (XLSX)
